# Clinical Utility of Stepwise Bronchoalveolar Lavage Fluid Analysis in Diagnosing and Managing Lung Infiltrates in Leukemia/Lymphoma Patients With Febrile Neutropenia

**DOI:** 10.1200/GO.23.00292

**Published:** 2024-02-01

**Authors:** Jayashree Thorat, Surabhi Bhat, Manju Sengar, Akshay Baheti, Sweta Bothra, Maheema Bhaskar, Sandeep Prakashnarain Tandon, Sanjay K. Biswas, Gaurav V. Salunke, George Karimundackal, Virendra Kumar Tiwari, C.S. Pramesh, Neha Sharma, Venkatesh Kapu, Thomas Eipe, Bhausaheb Pandurang Bagal, Lingaraj Nayak, Avinash Bonda, Amit Janu, Alok Shetty, Hasmukh Jain

**Affiliations:** ^1^ Department of Medical Oncology, Tata Memorial Centre, Affiliated with Homi Bhabha National University, Mumbai, India; ^2^ Hematological Cancer Consortium, Tata Memorial Centre, Affiliated with Homi Bhabha National University, Mumbai, India; ^3^ Department of Radio-diagnosis, Tata Memorial Centre, Affiliated with Homi Bhabha National University, Mumbai, India; ^4^ Department of Pulmonary Medicine, Tata Memorial Centre, Affiliated with Homi Bhabha National University, Mumbai, India; ^5^ Department of Microbiology, Tata Memorial Centre, Affiliated with Homi Bhabha National University, Mumbai, India; ^6^ Department of Surgical Oncology, Nanavati Hospital, Mumbai, India; ^7^ Department of Thoracic Surgical Oncology, Tata Memorial Centre, Affiliated with Homi Bhabha National University, Mumbai, India; ^8^ Department of Surgical Oncology, Tata Memorial Centre, Affiliated with Homi Bhabha National University, Mumbai, India; ^9^ Department of Clinical Pharmacology, Tata Memorial Centre, Affiliated with Homi Bhabha National University, Mumbai, India

## Abstract

**PURPOSE:**

Febrile neutropenia (FN) is a serious complication in hematologic malignancies, and lung infiltrates (LIs) remain a significant concern. An accurate microbiological diagnosis is crucial but difficult to establish. To address this, we analyzed the utility of a standardized method for performing bronchoalveolar lavage (BAL) along with a two-step strategy for the analysis of BAL fluid.

**PATIENTS AND METHODS:**

This prospective observational study was conducted at a tertiary cancer center from November 2018 to June 2020. Patients age 15 years and older with confirmed leukemia or lymphomas undergoing chemotherapy, with presence of FN, and LIs observed on imaging were enrolled.

**RESULTS:**

Among the 122 enrolled patients, successful BAL was performed in 83.6% of cases. The study used a two-step analysis of BAL fluid, resulting in a diagnostic yield of 74.5%. Furthermore, antimicrobial therapy was modified in 63.9% of patients on the basis of BAL reports, and this population demonstrated a higher response rate (63% *v* 45%; *P* = .063).

**CONCLUSION:**

Our study demonstrates that a two-step BAL fluid analysis is safe and clinically beneficial to establish an accurate microbiological diagnosis. Given the crucial impact of diagnostic delays on mortality in hematologic malignancy patients with FN, early BAL studies should be performed to enable prompt and specific diagnosis, allowing for appropriate treatment modifications.

## INTRODUCTION

Febrile neutropenia (FN) is a notable chemotherapy-related complication in hematologic malignancies.^1^ With the availability of broad-spectrum antibiotics, the mortality rates of patients with FN have diminished steadily but remains an important cause of morbidity and mortality.^2^ It is observed that lung infiltrates (LIs) are observed in about 30% of patients with FN.^3^ These patients have a high mortality rate, varying between 25% and 80%.^4,5^

**CONTEXT**

**Key Objective**
To evaluate the utility of performing a standardized bronchoalveolar lavage (BAL) fluid analysis for patients with leukemia and lymphoma presenting with febrile neutropenia (FN) and lung infiltrates.
**Knowledge Generated**
Our two-step BAL analysis exhibited a diagnostic success rate of 74.5%, surpassing most existing literature studies. This superior performance can be attributed to the implementation of a standardized BAL analysis procedure, which includes a panel of polymerase chain reaction tests. Notably, modifying antimicrobial treatment on the basis of BAL reports resulted in a significant improvement in clinical responses compared with nonmodified regimens.
**Relevance**
The challenge of obtaining accurate microbiological diagnoses in FN patients remains, and our results hold considerable importance for this specific group, particularly individuals with hematological malignancies. This standardized BAL technique was feasible in 83% of patients, demonstrating its practicality. This could help reduce unnecessary antimicrobial use, especially in areas with high drug resistance and limited health care resources.

One of the challenges in patients with FN with LIs is to accurately diagnose the underlying microbiological cause, which is possible in only 30% of patients with blood culture and serum galactomannan.^6^ Studies have shown that bronchoalveolar lavage (BAL) can improve the microbiological yield to 50%-65%.^6-8^ However, the battery of microbiological tests done in BAL fluid analysis is not uniform,^9,10^ and most centers use a combination of tests, including a fixed panel of polymerase chain reaction (PCR). An accurate microbiological diagnosis of LI is crucial for the judicious use of antibiotics to improve patient outcomes, and prevent antibiotic resistance and drug toxicity.

We developed a standardized procedure to conduct BAL and a two-step approach for the analysis of BAL fluid (Appendix 1). We aimed to evaluate the clinical utility and safety of this strategy in our patients with hematologic malignancies who presented with FN and LIs.

## PATIENTS AND METHODS

### Study Design and Setting

This prospective observational study was conducted at a tertiary cancer center in India, between November 2018 and June 2020. Patients age 15 years and older who met the following criteria were included in the study: a confirmed diagnosis of leukemia or lymphomas and undergoing chemotherapy, presence of FN, and evidence of LIs observed on chest X-ray or computed tomography chest imaging. Neutropenic fever was defined as a single oral temperature ≥101°F (38.3°C) or a temperature ≥100.4°F (38°C) for at least an hour, with an absolute neutrophilic count of <500 cells/mm^3^ or those with functional neutropenia with a predicted decline to ≤500 cells/mm^3^ over the next 48 hours.^3^

### End Points

The primary end point was to study the utility of using a two-step BAL analysis to identify the proportion of patients with a confirmed microbiological diagnosis (an isolate was considered as the causal agent as per the criteria proposed by Maschmeyer et al)^11^; for the diagnosis of invasive mycosis, the European Organisation for Research and Treatment of Cancer (EORTC)/Invasive Fungal Infections Cooperative Group and the National Institute of Allergy and Infectious Diseases Mycoses Study Group (MSG) criteria were used.^12^

The secondary end points were to evaluate the following: (1) the proportion of patients who had a change in antimicrobial therapy (on the basis of the BAL reports); (2) the feasibility of performing a bronchoscopy in this population; (3) the safety of BAL procedure (proportion of patients who develop major or minor complications)^13^; and (4) clinical and radiologic outcomes at 4 and 12 weeks.^14^

### Data Collection

The baseline demographic, disease, and treatment-related details were recorded, including current symptoms, symptom duration, examination findings, and the presence of a focus. Data on the duration of neutropenia, blood counts, culture reports, serum galactomannan levels, antibiotic selection, and modification to treatment were also noted. Before study enrollment, all patients were initiated on empirical antibiotics, either with or without antifungals, to address their FN. The selection and duration of antimicrobial treatments were individualized on the basis of each patient's clinical condition and their response to therapy, in accordance with the unit's policy and European Conference on Infections in Leukemia guidelines.^15^ BAL procedure and fluid analysis was performed using a standard protocol^13^ (Appendix 1). The samples were tested in the first stage for gram stain and aerobic culture, Ziehl-Neelsen stain, mycobacterial culture, calcofluor-white stain and fungal culture, galactomannan (enzyme-linked immunosorbent assay), and tuberculosis (TB) GeneXpert. If initial test results were negative, a stored BAL sample (ethylenediamine tetra acetic acid tubes at 4°C-8°C) was sent for PCR testing according to an algorithm dictated by radiologic pattern and infiltrates (Appendix 1).^14^ Modifications to the antimicrobial treatment on the basis of BAL reports were at the discretion of the clinician and recorded. All patients were followed up throughout the course of FN and to 12 weeks from enrollment. Complications within 24 hours of the procedure,^13^ as well as 4-week and 12-week outcomes (eg, clinical, radiologic, and survival outcomes), were noted. Radiology reviews were performed by two independent radiologists (A.J. and A.B.) and the results were categorized as stable, partial response, complete response, or progressive disease on the basis of the MSG and EORTC Consensus Criteria.^12,16^

### Statistical Plan

The estimated sample size was approximately 122 patients, which was necessary to determine a 50% incidence of positivity (range, 40%-60%) with a 5% margin of error at a 95% CI. This was calculated on the basis of previous studies in which the yield of BAL was 51% with a 95% CI of 26 to 76.^8,17^ Descriptive statistics and proportions were used to summarize epidemiologic data. The relationship between change in antimicrobials and final outcome was also analyzed using a chi-square test or Fisher's exact test. All statistical analyses were performed using the Statistical Package for the Social Sciences (IBM Corp Released 2012; IBM SPSS Statistics for Windows, version 21.0, IBM Corp, Armonk, NY).

### Ethics Approval

The study was approved by the institutional ethics committee and was conducted in accordance with Good Clinical Practice guidelines and the Declaration of Helsinki. The study was registered in the Clinical Trials Registry—India (CTRI/2018/10/016073).

### Consent

Patients were enrolled after obtaining a written informed consent. For patients age 15-17 years, assent and guardian consent were taken.

## RESULTS

**FIG 1:**
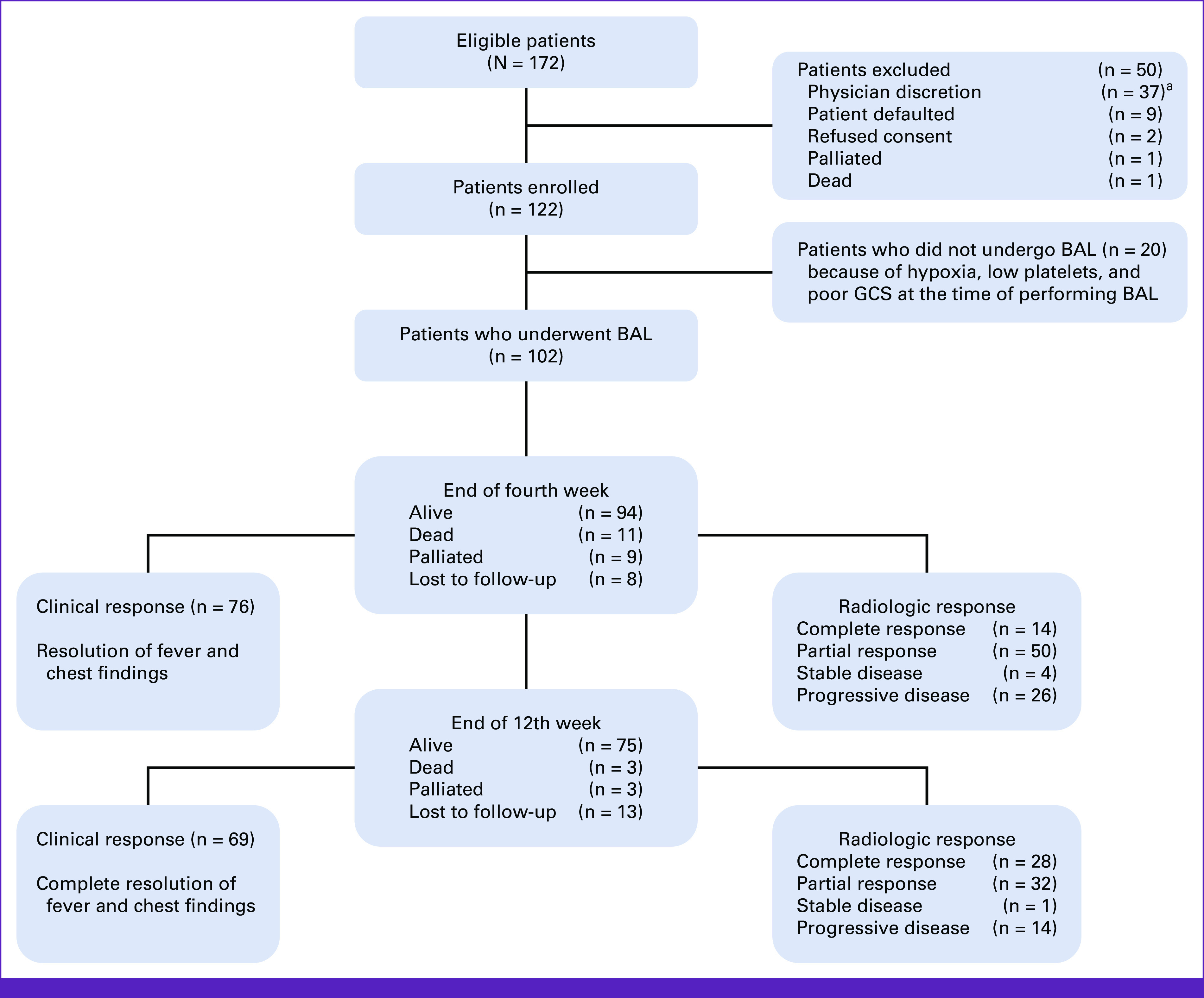
Baseline screening and enrollment of 122 patients. Clinical, radiologic, and survival outcomes of 102 patients who underwent BAL procedure at the end of fourth week and 12th week. ^a^Reasons that characterize physician discretion (n = 37) are BAL procedure deferred by the treating physician (n = 22) because of nondefinitive findings on CT scan and/or having clinical improvement on empirical treatment, definitive organism isolated from blood culture (n = 1), severe grade 4 thrombocytopenia (n = 11), hemodynamically unstable (n = 1), and referred to another center (n = 2). BAL, bronchoalveolar lavage; CT, computed tomography; GCS, Glasgow Coma Scale.

A total of 172 patients were eligible for the study, with 122 patients enrolled, as shown in Figure 1. The baseline characteristics of the patients are outlined in Table 1. The median age of the patients was 30 years (range, 15-65 years), with 70% male gender (n = 85/122). Acute leukemia was the primary diagnosis for 92.6% of the patients (n = 113). A total of 76 patients (62%) had an Eastern Cooperative Oncology Group performance status of 1 at baseline, and the median duration to BAL procedure from the time of presentation was 6 days (IQR, 3-10). Twenty patients (16.4%) were unable to undergo BAL analysis because of various complications, including hypoxia, low platelet count, and poor Glasgow Coma Scale scores. Eventually, BAL was successfully performed in 83.6% (n = 102/122) of the total patients, as illustrated in Figure 1.

**TABLE 1:** Baseline Characteristics of 122 Enrolled Patients

Characteristic	No. (%)
Age, years, median (range)	30 (15-65)
Hematologic malignancy	
ALL	43 (35.2)
AML	54 (44.3)
APML	16 (13.1)
Chronic lymphoproliferative/myeloproliferative disease (CLL, HCL, CML, and low-grade NHL)	5 (4.1)
High-grade NHL (B or T cell)	4 (3.3)
Performance status (ECOG)	
1	76 (62.3)
2	37 (30.4)
3	8 (6.5)
4	1 (0.8)
Comorbidities	
None	104 (85.2)
Diabetes mellitus	7 (5.7)
Hypertension	5 (4.1)
Hypothyroidism	5 (4.1)
Coronary artery disease	1 (0.8)
Concomitant tuberculosis	3 (2.4)
Bronchial asthma	1 (0.8)
Hematologic parameters (of 102 patients who underwent BAL)	
Hemoglobin, g/dL, mean (SD)	7.60 (±1.11)
Platelet count, ×10^9^ cells/L, median (minimum-maximum)	50 (19-584)
Absolute neutrophil count, cells/mm^3^, median	473
Severe neutropenia, ANC ≤500 cells/mm^3^	51 (50)
Profound neutropenia, ANC ≤100 cells/mm^3^	20 (19.6)

Abbreviations: ANC, absolute neutrophil count; APML, acute promyelocytic leukemia; BAL, bronchoalveilar lavage; CLL, chronic lymphocytic leukemia; CML, chronic myeloid leukemia; ECOG, Eastern Cooperative Oncology Group; HCL, hairy cell leukemia; NHL, non-Hodgkin lymphoma; SD, standard deviation.

Microbiological results are outlined in Table 2, which indicates that 26.5% of patients (n = 27/102) had bacterial isolates, including predominantly *Klebsiella pneumonia*, *Pseudomonas sp.*, and group A beta hemolytic *Streptococcus*. Tuberculosis was diagnosed in 6.9% of patients (n = 7/102) by GeneXpert, and fungal isolates were seen in seven patients, predominantly *Aspergillus* species. PCR results were sent for 22 patients, of whom 13 had a positive result, predominantly *Klebsiella pneumonia*, followed by *Acinetobacter baumannii*, as depicted in Table 2. The specific tests performed by PCR are provided in Appendix 1. BAL Galactomannan alone was positive in 43.1% of patients (n = 44/102). The initial analysis of BAL sample resulted a positive yield in 61.7% of patients (n = 63/102). Finally, with the addition of PCR testing, the yield was increased to 74.5% of patients (n = 76/102). Among the 122 patients included in the study, 20 individuals (16.4%) were documented to have bloodstream infections. Within this subgroup, five patients exhibited positive cultures in BAL samples. However, the microorganisms isolated from the bloodstream infections differed from those identified in the BAL cultures, highlighting a lack of correlation between these two clinical parameters.

**TABLE 2:** Microbiological Spectrum in BAL Fluid Analysis

Organism	Frequency, No.
Bacterial isolates (n = 34)	
*Klebsiella pneumoniae*	10
*Pseudomonas aeruginosa*	5
*Escherichia coli*	2
*Acinetobacter baumannii*	5
*Enterobacter aerogenes*	1
*Enterococci sp.*	1
Group A beta hemolytic *Streptococcus*	7
*Stenotrophomonas maltophilia*	1
*Citrobacter freundii*	1
*Proteus mirabilis*	1
*Mycobacterium tuberculosis* ^a^	7
Fungal isolates (n = 7)	
*Aspergillus* species	6
*Candida* species	1
Positive Aspergillus galactomannan in BAL samples (≥1)	44
Organism in PCR samples (n = 16)	
*Nocardia sp.*	1
Nontubercular *Mycobacterium sp*.	2
*Pneumocystis carinii pneumonia*	3
*Acinetobacter sp.*	3
*Klebsiella sp.*	4
Fungal sp.	3

NOTE. The table denotes all the positive microbiological samples obtained from 102 patients who underwent BAL analysis. 76 patients had a positive microbiological result, of whom 32 had more than one isolate. Among the patients who yielded positive cultures from BAL, five had documented bloodstream infections also, which were distinct from BAL results, with no correlation.

Abbreviations: BAL, bronchoalveolar lavage; PCR, polymerase chain reaction.

^a^ All instances of *Mycobacterium tuberculosis* detected were achieved by GeneXpert.

### Complications of BAL

Only one patient experienced a major complication, viz. persistent hypoxia (grade 2). Minor complications (hypoxia, hypertension, tachycardia, fever, bleeds, and hypertension) were recorded in 27 of 102 patients (26.4%) during the procedure and lasted up to 24 hours after the procedure in 21 of 102 patients (20.5%). However, all the patients recovered from these complications.

### Modifications in Antimicrobial Treatment

All patients who underwent the BAL procedure were already on antibiotics with or without antifungals, and modifications (addition/removal) in antimicrobial therapy on the basis of BAL reports were done for 63.9% of patients (n = 78/122) as depicted in Table 3. Among these patients, 53.8% (n = 42) had removal of an antibacterial drug, of whom 74% (n = 31) had sustained clinical and radiologic response at the end of 12 weeks. 14.1% of patients (n = 11) had removal of an antifungal drug, of whom 83% (n = 9) had sustained clinical and radiologic response at 12 weeks.

**TABLE 3:** Modification in Antimicrobial Therapy for 78 Patients

Change of Drug	Response at Fourth Week	Response at 12th Week
Removal of antibacterial drug in 42 patients (53.8%)	36 had clinical response and 34 had radiologic response Four died and two lost to follow-up	31 patients had sustained clinical and radiologic response Two died, two lost to follow-up, and one progressive disease
Removal of antifungal drug in 11 patients (14.1%)	Nine had clinical and radiologic response by the end of 4 weeks One died and one nonresponder	Nine patients had sustained clinical and radiologic response One nonresponder

NOTE. A modification of antimicrobial on the basis of BAL (addition and removal of antimicrobial) was done for 78 patients (63.8%), of whom 42 had removal of antibacterial and 11 patients had removal of antifungals, the outcomes of which are described in the table.

Abbreviation: BAL, bronchoalveolar lavage.

At the end of 12 weeks, 75 patients were alive, three died, three palliated, and 13 patients were lost to follow-up. The study showed that among patients who had a change in antimicrobial drugs, response was seen in 63% (n = 49/78) of patients compared with 45% (n = 20/44) of patients in whom there was no change in antimicrobials, resulting in a meaningful difference of 18% (*P* = .063). Clinical, radiologic, and survival outcomes are depicted in Figure 1.

## DISCUSSION

Pulmonary complications frequently occur in patients with hematologic malignancies and those who have undergone stem-cell transplantation. In these acutely ill patients, who have received myelosuppressive chemotherapy, achieving an accurate and early diagnosis is crucial.^18^ In our study, we assessed the feasibility and utility of a two-step approach of BAL fluid analysis in clinical practice in patients with FN and LIs. Across numerous studies involving BAL, acute leukemia consistently emerges as the most prevalent diagnosis, similar to our study population.^8,19,20^ The feasibility of BAL can be affected because of various factors such as risk of bleeding, hypoxia, and other complications. Owing to the retrospective nature of most of the studies reported in the literature, the feasibility in clinical practice could not be accurately determined. In our study, BAL was feasible in 83% of cases, which is similar to other prospective studies reported in this population.^8,17^

The diagnosis of LIs plays a pivotal role in determining the outcome of hematologic malignancies in patients presenting with FN.^21^ In our study, we found an enhanced diagnostic yield by using BAL fluid analysis for identifying probable bacterial and fungal pneumonia. Using conventional methods of microbial analysis, our yield was 61.7% (63/102). The addition of PCR testing to BAL culture-negative patients led to a further 13% increase in diagnostic yield, bringing the overall diagnostic yield to 74.5% (76/102). Comparative analysis of various prospective and retrospective studies (Table 4) shows similar rates of yield, adverse events, and treatment modification in the context of BAL utilization within a similar population.^8,17,20,22-28^ Khalid et al^22^ conducted a retrospective study to study the diagnostic yield of BAL in a similar population and reported 44% positivity. Similarly, an analysis of seven prospective and 16 retrospective studies reported a diagnostic yield with BAL fluid analysis to vary widely between 26% and 69%.^28^ It is noteworthy that our study reports higher diagnostic yield compared with others, which can be attributed to the utilization of a standardized BAL procedure (Appendix 1) and a well-defined microbiological testing algorithm (Appendix 1). According to the Maschmeyer criteria,^11^ the most prevalent etiology of pneumonia in our study was fungal (33%), followed by bacterial (28%), with PCR contributing an additional 13% positivity. Further studies on the etiology of pneumonia are necessary to improve antibiotic stewardship and establish their relevance. The microbiological spectrum may vary because of factors such as high local prevalence of tuberculosis, nosocomial pathogens, and other influencing factors. The two-step BAL approach at our institute led to efficient utilization of resources and improved patient outcomes. India being highly endemic for tuberculosis, TB GeneXpert testing was performed as part of the initial investigation, leading to the diagnosis of tuberculosis in seven (6%) patients at baseline.

**TABLE 4:** Comparison of the Utility and Safety of BAL in Patients With Hematologic Malignancies Across Multiple Studies

No.	Study	Type of Study and Time Period	Region	Age Group, Years	Cancer Diagnosis	Neutropenia Status	Yield, No. (%)	Adverse Events, %	Tests Performed on BAL	Treatment Modification After BAL, %
1	Khalid et al^22^	Retrospective 2015-2018	Pakistan	Mean: 26 ± 18	Hematologic and solid	Febrile neutropenia	40/90 (44%)	8.8	^a^	56.7
2	Hummel et al^20^	Retrospective 1988-2003	Germany	Median: 61 (range, 16-91)	Hematologic	During and outside neutropenia	47.9%	1.2	^b^	39
3	Svensson et al^23^	Retrospective 2004-2013	Sweden	Median: 57 (range, 19-84)	Hematologic	Neutropenic and non-neutropenic	59/151 (39%)	13	^c^	25
4	Boersma et al^8^	Prospective 2003-2005	The Netherlands	Median: 58 (range, 21-74)	Hematologic	Neutropenic patients	9/35 (26%)	<1	^d^	NA
5	Gilbert et al^24^	Retrospective 1996-2009	United States	Mean: 57.2 ± 11.3	Hematologic	Neutropenic and non-neutropenic	52.5%	—	^e^	44
6	Hohenadel et al^25^	Retrospective 4 years and 2 months	Germany	Median: 49.4 ± 14.5	Hematologic	Neutropenic and non-neutropenic	29/95 (31%)	15	^f^	84
7	Marchesi et al^17^	Prospective January 2018-September 2018	Italy	Median: 61 (range, 1-83)	Hematologic	Neutropenic patients	111/145 (76%)	<1	^g^	Approximately 50
8	Seneviratna et al^26^	Retrospective 2008-2009	New Zealand	Median: 50 (IQR, 43-68)	Hematologic	Febrile neutropenia	23%	7.7	^h^	38.4
9	Kim et al^27^	Retrospective 2009-2012	South Korea	Median: 50 (range, 17-78)	Hematologic	Neutropenic and non-neutropenic	65%	—	^i^	30.1
10	Our study	Prospective	India	Median: 30 (range, 15-65)	Hematologic	Febrile neutropenia	74.5%	26.4^j^	^k^	63.8

Abbreviations: AFB, acid-fast bacillus; BAL, bronchoalveolar lavage; CMV, cytomegalovirus; ELISA, enzyme-linked immunosorbent assay; HHV-6, human herpesvirus 6; HSV, herpes simplex virus; NA, not available; PCR, polymerase chain reaction; PJP, *Pneumocystis jirovecii* pneumonia; RSV, respiratory syncytial virus.

^a^ Gram staining, bacterial and fungal cultures, *Mycobacterium tuberculosis* culture and PCR, silver staining for PJP, and AFB staining for *Nocardia*.

^b^ Pappenheim, Gram, Ziehl-Neelsen staining, bacterial and fungal cultures, *Mycobacterium tuberculosis* culture, fungal DNA PCR, *Legionella pneumophila* culture, direct immunofluorescence test for *Chlamydia pneumoniae*, silver staining for PJP, ELISA for *Mycoplasma* and *Influenza A*, and serology for HHV-6, CMV, HSV, RSV.

^c^ Bacterial and fungal culture, PCR and immunofluorescence for respiratory virus, *Legionella*, PJP, *Mycobacteria* microscopy, PCR and culture, virus culture; immunocompromised—CMV PCR and antigen, HSV PCR, *Aspergillus* PCR, *Pneumococci* PCR, *Mycoplasma* and *Chlamydophila* PCR.

^d^ Gram staining and Ziehl-Neelsen staining, bacterial culture, mycobacterial culture, *Legionella* culture, and viral culture (check for fungal).

^e^ Bacterial culture, fungal culture, viral culture, smear, and mycobacterial culture.

^f^ Gram, acridine, and acid-fast stains, cultured for bacteria, fungi, mycobacteria, and viruses, culture and PCR for *Legionella*, and immunofluorescence for CMV.

^g^ Bacterial and fungal cultures, cytologic examination, galactomannan, PCR for respiratory bacteria and viruses, CMV, PCR, and immunofluorescence for PJP, and PCR for *Mycobacterium tuberculosis* and *Aspergillus spp*.

^h^ Bacterial cultures, viral cultures and immunofluorescence, and PJP immunofluorescence staining.

^i^ Gram and Ziehl-Neelsen staining, bacterial, mycobacterial, and fungal culture, silver staining and immunofluorescence test for PJP, immunostaining for CMV, direct immunofluorescence for respiratory viruses, PCR for CMV, and PJP and mycobacteria.

^j^ Complications were hypoxia, hypertension, tachycardia, fever, bleeds, and hypertension, and all patients recovered.

^k^ Gram stain and bacterial cultures, fungal stain and cultures, Ziehl-Neelsen stain and *Mycobacterium* cultures, galactomannan and PCR for bacteria, viruses—CMV, *Adenovirus*, HHV6, *Influenza*, *Nocardia*, *Pneumocystis jirovecii*, *Aspergillus*, *Legionella*, *Mucor*, *Mycoplasma*, *P. jirovecii*, *Mycobacterium TB*, atypical *Mycobacterium TB* (on the basis of the diagnostic algorithm).

A total of 78 patients (63.9%) underwent modifications in their antimicrobial therapy, resulting in considerable improvements in their clinical outcomes. These findings underscore the valuable role of using accurate diagnostic approaches to facilitate timely and evidence-based initiation, change, or discontinuation of antimicrobial and antifungal treatments in patients. This practice not only reduces health care costs and unnecessary antimicrobial use but also alleviates the burden on health care facilities and contributes to the mitigation of antimicrobial resistance. Several other studies have reported similar modification rates, ranging from 38.2% to 63.3%, with effective outcomes in a majority of patients.^20,22,28^ The impact of BAL on reducing unnecessary antimicrobial use, especially in regions with high drug resistance and limited resources, is important without affecting response rates. We observed that change in antimicrobial therapy resulted in a 17.3% (*P* = .063) increase in clinical response compared with no change in therapy, demonstrating the high clinical relevance of BAL findings.

In our study, BAL was associated with minor complications, and no grade 3 events were reported, aligning with findings from previous studies. These results reinforce the safety of the BAL procedure in a high-volume setting.^23,26,28^ Our study has a few limitations. The two-step approach used in our study may cause delays, particularly in patients with negative blood and BAL cultures, where PCR analysis was conducted subsequently. Another limitation was the use of empirical antibiotics before the BAL analysis, which may have influenced the yield.

In summary, our study results are applicable to high-volume settings treating leukemia and lymphoma, as well as populations with a high incidence of fungal infections. Our findings also demonstrate improved diagnostic yields through the use of standardized BAL procedures, a consistent microbiological algorithm, and PCR testing. The yield of standard BAL procedures set a benchmark, which may be improved by incorporating whole genome–based next-generation sequencing methods, which shows promise in enhancing culture-independent pathogen screening.^29^

In conclusion, our study showed that the two-step BAL fluid analysis is a safe, clinically relevant, and cost-effective strategy that can detect a potential cause of LIs in almost three fourths of patients with hematologic malignancies and FN. Thus, considering the high impact of diagnostic delay on mortality in immunocompromised patients, BAL studies should be performed early in course of disease to obtain specific diagnosis and make changes in treatment accordingly.

## Data Availability

Trial data will be made available on request by writing an e-mail to the corresponding author for the purpose of systematic reviews and meta-analyses. The data will be shared after institutional ethics committee approval of the proposal and regulatory clearances (Department of Atomic Energy).

## APPENDIX 1. STEPWISE ALGORITHM FOR ANALYSIS OF BAL FLUID

### Prerequisites for Bronchoalveolar Lavage Procedure

Patients will be nil per oral at least 6 hours before the procedure and hemodynamically stable, maintaining oxygen saturation more than 90% with or without supplemental oxygen using nasal cannula and platelet count should be more than 30,000 cells/mm^3^ with no active bleeding diathesis.

#### Equipment

1. Flexible bronchoscope sterile collection trap (three per patient)
2. Suction tubing
3. Sterile warm saline
4. Vacuum source
5. Syringe 20 mL, 10 mL, 50 mL
6. Optional: three way stop-cock
7. Lidocaine gel, 2%, 4% solution, and spray
8. Midazolam
9. Flumazenil (five ampoules in stock)
10. Oxygen cannula

#### Preprocedure

1. The subject has been fasting for >6 hours for solid food and >4 hours for clear fluids
2. Preprocedure observations are recorded, including blood pressure (BP), heart rate, respiratory rate (RR), oxygen saturation, and the need for oxygen in litres per minute if required
3. Obtain informed consent
4. If an outpatient procedure, the patient should be accompanied by a person designated to drive the patient
5. Bronchoalveolar lavage (BAL) should be planned to be performed before any other bronchoscopic procedure
6. Review radiographs to determine ideal site of alveolar lavage
  - In diffuse infiltrates, the right middle lobe or the lingula in the supine patient is preferred
7. Monitoring equipment is attached, including three-lead ECG, pulse oximeter, and sphygmomanometer. Anesthetic backup will be required
8. Oxygen may be delivered via nasal cannula at up to 4 L/min, to achieve saturation >90%
9. The operator checks the collection trap, and tubing and bronchoscope, and ensures effective suction by aspirating sterile normal saline
10. Bronchodilators as indicated in patients with obstructive airway disease
11. For sedation, if appropriate and needed, intravenous midazolam is administered (adult dose 1-2.5 mg IV) and amount is recorded
12. Resuscitation equipment should be readily available
13. A small gauge IV cannula for medications is inserted

#### Procedure

1. Scope introduction is usually via the nose. If nasal introduction is not possible because of polyps or inflamed turbinates, oral approach is used with mouth guard to prevent damage to patient's teeth or scope
2. Topical anesthesia at the larynx is completed using 4% lidocaine; usually a total of 4-6 mL is used. Commonly, this causes coughing on instillation
3. The vocal cords are passed, and further mucosal anesthesia using 2-mL aliquots of 2% lidocaine at the carina, at the division of the lobes (proposed maximum limits vary from 4 to 5 mg/kg). Minimize use of topical anaesthesia as there may be bacteriostatic effects of lidocaine (use only the necessary amount to minimize coughing, and document the total amount of lidocaine used in the procedure)
  - Avoid suctioning before obtaining BAL specimen
  - If needed, however, the suction channel should be thoroughly rinsed with saline before BAL
4. Advance bronchoscope until wedged in a desired subsegmental bronchus at the desired location
  - Avoidance of bronchial trauma is particularly important in the patient with suspected alveolar hemorrhage
5. The bronchoscope is positioned within the lobe/segment but not too distal so that the airway collapses when suction is applied (Wink test using the suction button)
6. Syringes are prefilled with warmed normal saline
7. When the subject and the bronchoscopist are ready, the first syringe of saline is instilled by the bronchoscopy assistant, while the bronchoscopist maintains the position
8. Infuse 20 mL of saline with a syringe, observing the flow of saline at the distal tip of the bronchoscope. After each aliquot, flush channel with 5 mL air to clear the channel
9. Maintaining wedge position, apply gentle suction (50-80 mmHg), collecting the lavage specimen in the collection trap; if collapsing of segment is noted, then perform manual aspiration with 50 cc syringe
10. Repeat steps 5 and 6, up to five times as needed (total 100-120 mL), to obtain an adequate specimen (40-60 mL—usually 40%-70% recovery of total instillate)
  - Observe for flow of bubbles returning from the alveolar space
  - Gentle reorientation of bronchoscope tip may allow better return of fluid
  - Distal airways may collapse at higher negative suction pressures
  - Reduction in pressure or intermittent suctioning may help with distal airway collapse
  - Instructing the patient to inhale and exhale deeply may also help improve return of specimen
  - Higher aliquots and higher total volume can occasionally be used (up to 300 mL)
  - Note input and output of BAL
11. BAL specimen should be processed as soon as possible with desired tests ordered and transported on melting ice
12. Heart rate, BP, RR, saturation, and requirement of oxygen during procedure are recorded
13. The bronchoscope is slowly fully withdrawn

#### Subject Recovery

1. The subject is monitored for 2-4 hours in the recovery room
2. During this time, they are allowed to rest and then only eat and drink >120 minutes after the procedure (when their swallow is assessed as safe), to reduce the risk of aspiration
3. Postprocedural observations are monitored and recorded (heart rate, BP, RR, saturation, and requirement of oxygen ½ hourly for 2 hours) and the patient is examined clinically before discharge
4. The subject is briefed predischarge about the common side effects (fever, mild right chest discomfort, and sore throat) and given a contact number in case significant side effects occur
5. The subject is followed-up at 1-5 days after procedure with a clinical contact

### Microbiologic Workup Algorithm

Initially samples will be sent for

1. Gram stain and bacterial cultures
2. Fungal stain and cultures
3. Ziehl-Neelsen stain and *Mycobacterium* culture
4. Galactomannan

If all the above-mentioned tests come negative, then the stored BAL sample will be sent for polymerase chain reaction (PCR) testing according to given diagnostic algorithm.

### Diagnostic Algorithm

Appendix Table A1.

**TABLE A1:** Methodology and Diagnostic Algorithm for Identifying Atypical Bacteria, Viral, and Fungal Organisms in Pulmonary Infiltrates

Nodular Infiltrates	Diffuse/Micronodular Infiltrates
Bacteria	Viruses: CMV, *Adenovirus*, HHV6, *Influenza*
*Nocardia*	*Pneumocystis jirovecii*
*Aspergillus*	*Legionella*
*Mucor*	*Mycoplasma*
*P. jirovecii*	
*Mycobacterium TB*	
Atypical *Mycobacterium TB*	

Parameter	Methodology
Atypical MTB	Real-time PCR
Atypical bacteria—*Chlamydia*, *Mycoplasma*, *Legionella*	PCR and sequencing
*Pneumocystis jirovecii*	PCR
*Nocardia*	PCR
Viral influenza, parainfluenza, *rhinovirus*, *metapneumovirus*, RSV, *coronavirus*	Real-time PCR
Pan-fungal organisms	PCR and sequencing

Abbreviations: CMV, cytomegalovirus; HHV6, human herpesvirus 6; MTB, mycobacterium tuberculosis; PCR, polymerase chain reaction; RSV, respiratory syncytial virus; TB, tuberculosis.
